# 145. Comparing Antibiotic Use Across Inpatient Facilities with Different Antibiotic Stewardship Typologies using Machine Learning and Joint Modeling Approach

**DOI:** 10.1093/ofid/ofab466.347

**Published:** 2021-12-04

**Authors:** Yue Zhang, Jincheng Shen, Tina M Willson, Edward A Stenehjem, Tamar F Barlam, Mari-Lynn Drainoni, Ellen Childs, Jorie M Butler, Matthew B Goetz, Matthew B Goetz, Karl Madaras-Kelly, Reasdon Caitlin M., Matthew H Samore

**Affiliations:** 1 University of Utah, Salt Lake City, UT; 2 Intermountain Healthcare, Salt Lake City, UT; 3 Boston Medical Center, Boston, Massachusetts; 4 Boston University, Boston, Massachusetts; 5 VA Greater Los Angeles Healthcare System and David Geffen School of Medicine at UCLA, VA-CDC Practice-Based Research Network, Los Angeles, California; 6 Idaho State University, Pocatello, Idaho; 7 VA Center for Clinical Management Research, Ann Arbor, Michigan; 8 VA Salt Lake City Healthcare System, Salt Lake City, Utah

## Abstract

**Background:**

Hospital antibiotic stewardship programs (ASP) aim to promote the appropriate use of antimicrobials (including antibiotics) and play a critical role in controlling antibiotic costs and antibiotic-resistant bacterial infection risk, and improving patient outcomes. However, unlike other health care quality improvement intervention programs, the ASP implementation strategies vary among healthcare facilities, and little is known about whether different types of ASP implementation will lead to the shifting of antibiotic drug use from one class to another.

**Methods:**

We proposed an analytical framework using unsupervised machine learning and joint model approach to 1) develop a typology of ASP strategies in facilities from the Veterans Health Administration, America’s largest integrated health care system; and 2) simultaneously evaluate the impacts of different ASP types on the annual antibiotic use rates across multiple drug classes. The unsupervised machine learning method was used to leverage the structural components in the surveys conducted by the Veteran Affair (VA) Healthcare Analysis and Information group and the Consolidated Framework for Implementation Research experts from Boston University, and reveal the underlying ASP patterns in the VA facilities in 2016.

**Results:**

We identified 4 groups in the VA facilities in terms of enthusiasm and implementation level of antibiotic control in our ASP typology. We found the facilities with high implementation level and high enthusiasm in ASP and those with high implementation level but low enthusiasm had statistically significant 30% (p-value=0.002) and 22% (p-value=0.031) lower antibiotic use rates in broad-spectrum agents used for community infections, respectively than those with low implementation level and low enthusiasm. However, the facilities with high implementation and high enthusiasm also marginally increased antibiotic use rates in beta-lactam antibiotics (p-value=0.096).

**Conclusion:**

The developed analytical framework in the study provided an approach to the granular assessment of the impact of the healthcare intervention programs and might be informative for future health service policy development.

**Disclosures:**

**Matthew B. Goetz, MD**, Nothing to disclose

